# Multiparameter flow cytometric and transcriptional analyis of CD20 positive T-cells in bone marrow in patients of multiple myeloma and monoclonal gammopathy of undetermined significance

**DOI:** 10.3389/fimmu.2025.1464940

**Published:** 2025-02-26

**Authors:** Barbara Forró, Béla Kajtár, Ágnes Lacza, László Kereskai, Livia Vida, Balázs Kőszegi, Péter Urbán, József Kun, Attila Gyenesei, Szabolcs Kosztolányi, Dániel Kehl, Pál Jáksó

**Affiliations:** ^1^ Department of Pathology, University of Pécs Medical School, Clinical Centre, Pécs, Hungary; ^2^ Department of Biochemistry and Medical Chemistry, University of Pécs Medical School, Pécs, Hungary; ^3^ Genomics and Bioinformatics Core Facility, Szentágothai Research Centre of the University of Pécs, Pécs, Hungary; ^4^ Department of Pharmacology and Pharmacotherapy, University of Pécs Medical School, Pécs, Hungary; ^5^ 1st Department of Internal Medicine, University of Pécs Medical School, Pécs, Hungary; ^6^ Faculty of Business and Economics, University of Pécs, Pécs, Hungary

**Keywords:** multiple myeloma, CD20, T-cells, tumor immunology, T-cell exhaustion

## Abstract

**Introduction:**

CD20+ T-cells were described firstly in peripheral blood and later in bone marrow in patients with hematological tumors, and certain immune-mediated diseases. During our hematological diagnostic work, this peculiar subgroup of lymphocytes has been consistently observed associated with untreated monoclonal gammopathy of undetermined significance (MGUS) and myeloma (MM). Despite the expanding literature data, the exact function of CD20+ T cells remains unclear.

**Methods:**

We investigated the incidence of CD20+ T-cells in MGUS (n=27), and MM using a larger cohort (n=125) and compared it with control bone marrow samples (n=39). We examined their presence before and after treatment in 32 cases with flow cytometry. Comprehensive flow cytometric analysis included the examination of functional (T-cell activation, cytotoxic molecules and T-cell exhaustion) and maturation markers in a large number of cases. In addition RNA sequencing and subsequent bioinformatics analyses were carried out to detect differentially expressed (DE) genes of FACS sorted CD20+ T-cells versus CD20- T-cells.

**Results and discussion:**

We found that CD20+ T-cells are phenotypically and transcriptionally different from CD20- T-cells. Elevated incidence of CD20+ T-cells in MGUS and MM and the expression of CD8, NKG2D, and CD28 suggests anti-tumor functionality. Increased PD-1 expression indicates T-cell exhaustion which was mostly detected in the samples of patients with a higher tumor percentage. The majority of CD20+ T-cells are effector or effector memory T-cells. Some of the differentially expressed genes suggest antitumor function via regulating T-cell activation pathways, while other genes involved in tumor escape from immune surveillance by suppressing T-cells or by reprogramming T-cells toward T-cell exhaustion. Our findings suggest that CD20+ T-cells may play a vital role both in immune surveillance and immune escape contributing to progression of multiple myeloma.

## 1 Introduction

CD20 antigen is a transmembrane protein, previously described as a pan-B cell marker present on a broad variety of normal and neoplastic B-cells. A small subset of CD3+ T-cells was also found to express CD20, that was first reported by Hultin et al. in healthy peripheral blood (1). Algino et al. identified CD20 on T-cells in patients of acute lymphoblastic leukemia (ALL) and chronic lymphocytic leukemia (CLL) (2). Two latter reports revealed that in some occasional T-cell neoplasm cases CD20 appeared on T-cells as an abnormal phenomenon (3, 4). CD20 on T-cells was also seen in patients of rheumatoid arthritis, but this finding was considered as an artifact of flow cytometry (FCM) (5). Schuh et al. (6) described them in the thymus, bone marrow and secondary lymphoid organs and they were also found in cerebrospinal fluid, in patients with multiple sclerosis. De Bruyn et al. (7) described them as Tc1 effector memory T-cells in peritoneal ascites fluid with patients of ovarian cancer.

The following assumptions arise regarding the origin of CD20+ T-cells. According to a hypothesis, CD20 molecule on T-cells is a result of ex vivo storage of blood samples that leads to antigen exchange between T- and B-cells (8). Another approach is that CD20 expression on T-cells is a result of a specific acceptor cell mediated process, called trogocytosis or “shaving reaction”. During this molecular reorganization - also called ‘immunological synapse -’ T-cells could extract CD20 from the antigen presenting cells, eventually presenting it on their own surface (7, 9). On contrary, Schuh et al. (6) aimed to clarify these issues, therefore cell sorting experiments were carried out on CD3+/CD19-/CD20+, CD3+/CD19-/CD20- T-cells and CD3-/CD19+/CD20+/B-cells from peripheral blood of healthy individuals, and quantitative PCR was run on each population to compare them at the transcriptional level. As described, CD3+CD19-CD20+ cells themselves transcribe CD20.

During our diagnostic work on hematological malignancies, we noticed elevated percentages of CD20+ T-cells in patients of myelodysplastic syndrome (MDS), MGUS, and MM. The highest proportions were consequently observed in diagnostic MM samples, where in some occasional cases, the proportion of these cells reached 30-35% among lymphocytes. When these data were compared with control bone marrow ratios, we measured an average difference of 8-10% (n=10, 15-20% vs. 2-3%, respectively. We investigated the expression of NKG2D (CD314), a marker associated with responses to cellular distress, infections (10). In 9 out of 10 cases, our target cells were found to be positive for NKG2D, based on these findings, we assumed that these cells may participate in antitumor immune responses which, in turn could be crucial in achieving long term disease control in MM patients (11). Studies in the context of cancer are limited, therefore we set out to expand the data regarding CD20+T-cells in the bone marrow of MM cases, and to investigate if they have any impact on the course of the disease.

Based on the preliminary findings above, comprehensive flow cytometric studies were performed on CD20+ T-cells, their incidence and phenotype were examined on a larger cohort, consisting of MM, MGUS and control samples. Correlating with the literature (12), CD20+ T-cells showed mostly CD8 positive cytotoxic phenotype, so the flow cytometric part of the current study was restricted to CD20+CD8+ T-cells. Our panel contained four backbone markers, CD45, CD20, CD3, CD8 and functional markers such as CD28, CD137, CD25, CD103, NKG2D, perforin, granzyme-B, LAG-3 (CD223), PD-1(CD279). We also examined CD45RA and CCR7 expression to classify them into maturation categories (8, 13). To access the transcriptional profile, CD3+CD20+ and CD20- cells were sorted and analyzed using RNA sequencing. In addition, we monitored the proportion of CD20+ T-cells from the time of the diagnosis of MM, and through the follow-up of treatment and looked for a correlation between the CD20+ T-cells ratios and the proportion of measurable residual disease (MRD) of MM samples.

## 2 Materials and methods

### 2.1 Patients and samples

Samples and data were collected and handled with the authorization of the Regional Ethics Committee of University of Pécs. Bone marrow (BM) samples were collected from patients with confirmed diagnosis of MM (n=125) and MGUS (n=27) according to International Myeloma Working Group updated criteria for the diagnosis of Multiple Myeloma and Related Plasma Cell Disorders (14).

The demographic distribution MM and MGUS patients ranged from 42 year to 84 years with a mean age of 67. The patients with confirmed diagnosis of MM included 65 females and 60 male patients, whereas in MGUS, 15 belonged to female and 12 to male patients. Control samples derived from patients with presumptive clinical diagnosis of MM or other hematological disease that were not verified based on hematopathological investigations (n=16), and from patients being in complete remission following treatment of a hematological disorder (n=23). The demographic distribution of the control patients ranged from 1 year to 81 years. Among control samples without confirmed hematological malignancy, 9 belonged to female and 7 to male patients, with mean age of 74. The control patients in remission following therapy for non-myeloma hematological malignancies included 12 males and 11 females, with mean age of 31. In 32 MM patients, we could perform a follow up from the time of the diagnosis and during treatment. The measurable residual disease was detected by FCM and bone marrow core biopsy histology. Patients were treated with a proteasome inhibition-based triplet therapy and, whenever applicable, autologous stem cell transplantation was performed following ESMO Clinical Practice Guidelines (15).

### 2.2 Preparation of bone marrow samples for flow cytometry

**Figure 1:**
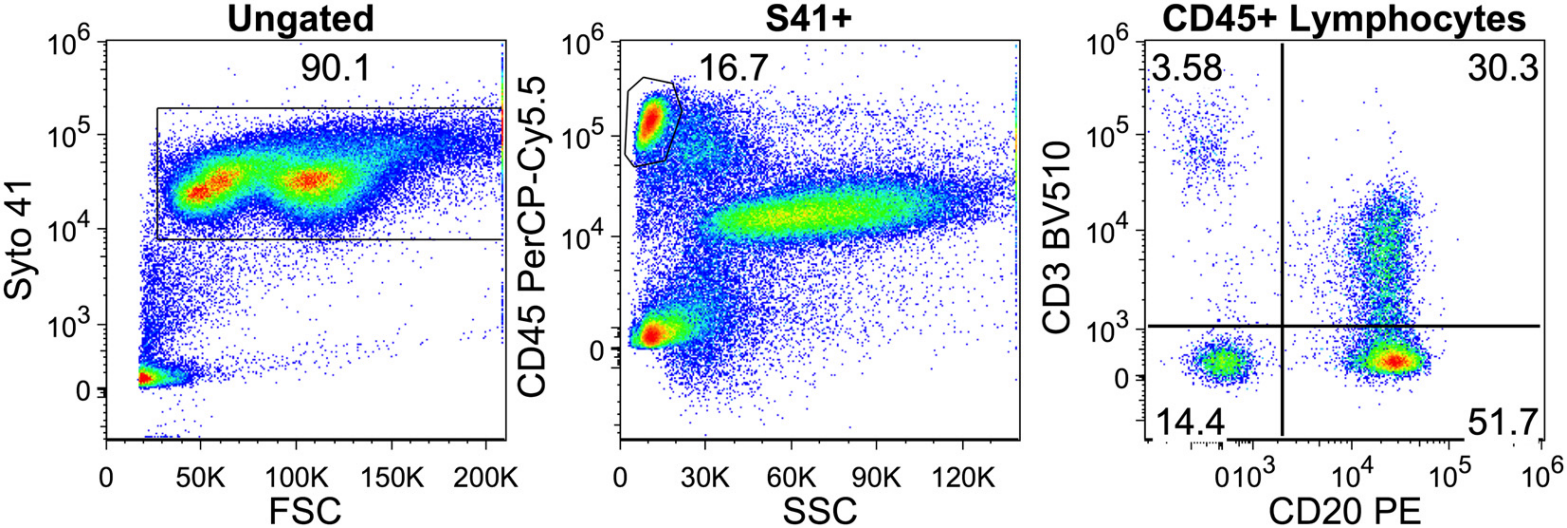
Gating on CD20+ T-cells in a multiple myeloma patient. Intact white blood cells were gated based on Syto 41 positivity and forward scatter. Lymphocytes were separated by CD45 and side scatter. Finally in the lymphocyte gate CD20+ T cells can be seen on the upper right quadrant.

Bone marrow aspirates were obtained from the iliac crest region and collected in BD Vacutainer ® EDTA tubes. Bone marrow cells (5-10x10^5^) were labeled with fluorescently conjugated antibodies and incubated for 15 minutes at room temperature, protected from light. Erythrocyte lysis and cell fixation was carried out according to the manufacturer’s recommendations, briefly Excellyse I lysing buffer (Exbio) was added for 2 minutes, and after dilution with DI water, incubated for another 10 minutes. Samples were spun down at room temperature, using 400xG for 5 minutes and the pellet was resuspended in 500 ml of phosphate-buffered saline (PBS). Finally, Syto 41 (S41) nucleic acid stain (ThermoFisher) was added and incubated for 2 minutes. In the case of perforin and granzyme-B, after labeling with backbone surface markers, intracellular staining protocol was performed following the guidelines of the manufacturer (Fix and Perm Cell Permeabilization Kit, ThermoFisher). 1x10^5^ S41 positive cells were collected using Navios Flow Cytometer (Beckman Coulter). Data were analyzed using Flowjo software (version 9.8.5.). We started the analysis by discriminating nucleated cells from debris using S41 and forward scatter (FSC) parameters. CD45 and side scatter (SSC) was used to identify lymphocytes and further divided into CD3 (clone: SK7, Sony) positive/negative and CD20 (clone: 2H7, Exbio) positive/negative lymphocytes, according to **Figure 1**. Fluorescent dyes and manufacturers of the additional monoclonal antibodies for phenotyping are briefly: BB515-conjugated anti-CD279 (clone: EH12.1, BD Biosciences), PE-Cy7-conjugated anti CD28 (clone: CD28.2, Sony Biotechnology), APC-conjugated anti-CD314 or NKG2D (clone: BcR-A-CT35, Sony Biotechnology), BV510-conjugated anti-CD8 (clone: SK1, BD-Biosciences), FITC-conjugated Granzyme-B (clone: Gb 11, Beckman-Coulter), APC-conjugated anti-CD25 (clone: M-A251, Sony Biotechnology), APC-R700 anti-CD223 (clone: T47-530, BD Biosciences), APC-conjugated anti CD137 (clone: 4B4-1, Sony Biotechnology), FITC-conjugated Perforin (clone: delta G9, Miltenyi Biotec).

### 2.3 Isolation of bone marrow mononuclear cells by Ficoll-Paque technique and cell sorting by Fluorescence-Activated Cell Sorting (FACS)

Lymphocytes were isolated from bone marrow aspirate samples (n=3) by Ficoll-Paque density gradient centrifugation (Ficoll-Paque™ PLUS, GE Healthcare Bio-Sciences). All subsequent steps were performed on a laminar flow cabinet to prevent contamination. The Ficoll solution (1 ml) was placed in the lower fraction, and the bone marrow (2 ml) was layered on it by careful pipetting. After that, centrifugation took place for 20 minutes at 470xG. The layer of mononuclear cells were transferred into sterile 15 ml conical tube and washed with 10 ml of PBS and spun down for 5 min, 400xG. After discarding supernatant, we resuspended cells in 300 µl of cooled sterile PBS with 2% (v/v %) FBS (Fetal Bovine Serum, Gibco) and cells were proceeded to cell count. 1x10^7^ cells were stained with CD45 FITC (clone: HI30, Sony), CD3-ECD (Clone: UCHT1, Beckman Coulter), CD20-PE (clone: LT20, Exbio) fluorescent antibodies and incubated for 15 minutes, at 4°C, protected from light. After incubation, cells were washed and centrifuged with FBS-PBS (2%) and resuspended in 1ml of FBS-PBS (2%). Samples were sorted with SONY SH800 Cell Sorter (Sony Biotechnology). Debris and aggregates were eliminated based on FSC/SSC, lymphocytes were isolated based on CD45/SSC, and divided into two groups: CD3+CD20+ and CD3+CD20- cells. On average, approximately 1x10^5^ CD3+CD20+ cells and 3x10^5^ CD3+CD20- cells were sorted per patient.

### 2.4 RNA extraction of sorted CD3+CD20+ vs. CD3+CD20- T-cells

After sorting, extra FBS was added to the samples, adjusting 10 v/v % final volume. Samples (n=3) were spun down for 5 min, 400xG, and resuspended in 300 µl of Trizol (TRIzol® Reagent, Invitrogen by Thermo Fisher Scientific) and proceeded to RNA isolation. For RNA isolation, RNA isolation kit (Direct-zol™ RNA MicroPrep, Zymo Research) was used. RNA was air-dried and dissolved in double-distilled water (ddH2O) and devided in two portions for RNA-sequencing and real-time quantitative PCR.

### 2.5 3’ mRNA sequencing of CD3+CD20+ vs. CD3+CD20- T-cells

RNA libraries were generated using the QuantSeq 3’ mRNA-Seq Library Prep Kit FWD for Illumina (Lexogen). Briefly, 50 ng of total RNA was used as input for first strand cDNA generation using oligodT primer followed by RNA removal. Thereafter, the second strand synthesis is initiated by random priming and the products were purified with magnetic beads. Finally, the libraries were amplified and barcoded using PCR. All libraries were assessed on the Agilent 4200 TapeStation (Agilent Technologies) to examine if adapter dimers formed during PCR. The QuantSeq libraries were sequenced using the Illumina NovaSeq 6000 platform (Illumina Inc.) to produce 75 base pair single end reads. Binary Base Call (BCL) files generated by the sequencing instrument were base called, demultiplexed and translated into FASTQ files using bcl2fastq v2.20.0.422 software (Illumina Inc.). Reads were subsequently filtered for a minimum length of 40bp and quality-trimmed to Q30 using the Phred algorithm with BBDuk from the BBTools suite v38.86 (16). The processed reads were aligned against the human reference genome (GRCh37 Ensembl release) with STAR v2.7.6a (17). The number of reads aligned within each gene was counted using HTSeq Python library v0.11.1 (18). Gene count data were normalized using the trimmed mean of M values (TMM) normalization method of the edgeR R/Bioconductor package (v3.28, R v3.6.0, Bioconductor v3.9). Data were further log transformed using the voom approach for statistical evaluation in the limma package (19). Fold change (FC) values between the compared groups and moderated t-test p-values were calculated by the limma package. Normalized counts in exploratory data analysis and visualization were represented as transcripts per million (TPM) values.

### 2.6 Determination of CD20 (MS4A1) mRNA level by real-time quantitative PCR

Reverse transcription was processed by the High-Capacity cDNA Reverse Transcription Kit (Applied Biosystems) according to manufacturer’s instructions. The reaction mixture contained 50-100 ng of extracted cellular RNA. Real-time quantitative-PCR reaction mixture total volume was 25µl and contained the following components: 5µl cDNA, 4mM MgCl2, 400µM of each dNTP, 10X PCR buffer (Roche Molecular Biochemicals), 1 U FastStart Taq DNA polimerase (Roche Molecular Biochemicals), 0,3µM of forward-reverse primer mix, 0,2µM of PCR probe. The PCR conditions were set as follows: pre denaturation: 95°C, 6 min; 95°C, 35 sec; 59°C, 35 sec; 72°C, 35 sec; 45 cycles. The following primers and probes were used: human MS4A1 forward, ATGTCTTCACTGGTGGGCC; human MS4A1 reverse, TAATCTGGACAGCCCCCAA; human MS4A1 probe, [6FAM]CACGCAAAGCTTCTTCATGAGGGAATCT[TAM]; human CABL forward, TGGAGATAACACTCTAAGCATAACTAAAGGT; human CABL reverse, GATGTAGTTGCTTGGGACCCA; human CABL probe [6FAM]CCATTTTTGGTTTGGGCTTCACACCATT[TAM]. PCR was run on StepOnePlus™ Real Time PCR System (Life Tecnologies). Data were evaluated using 2-ΔΔCq method.

## 3 Results

### 3.1 Ratio of CD20+ T-cells in control, MGUS and MM groups

As a first step we analyzed differences between the control group and MGUS and MM patients according to their CD20+ T-cell ratios among lymphocytes and S41+ nucleated cells, respectively. In MGUS and MM samples the median ratio of CD20+ T-cells was roughly two times higher (10.3% and 12.3%, respectively) compared to control bone marrow samples (6.26%). The groups were compared using the Kruskal-Wallis’s rank sum test. In both cases, considering CD20+ T cell/lymphocyte ratios and CD20+ T-cells/S41+ cell ratios we found evidence of a difference in the distributions of the control vs. MGUS and MM groups (p-value < 0.001). We analyzed our data for correlation between presence of CD20+ T cells on age and gender for both control and MM/MGUS patients. We did find low positive but non-significant Pearson correlation between age and presence (control, r =0.279, p = 0.176, MM/MGUS, r = 0.06, p = 0.495). We did not find any evidence either in case of gender and presence (Mann-Whitney-Wilxocon, control W = 62.5, p = 0.4145, MM/MGUS, W = 3082, p = 0.1184).

**Figure 2:**
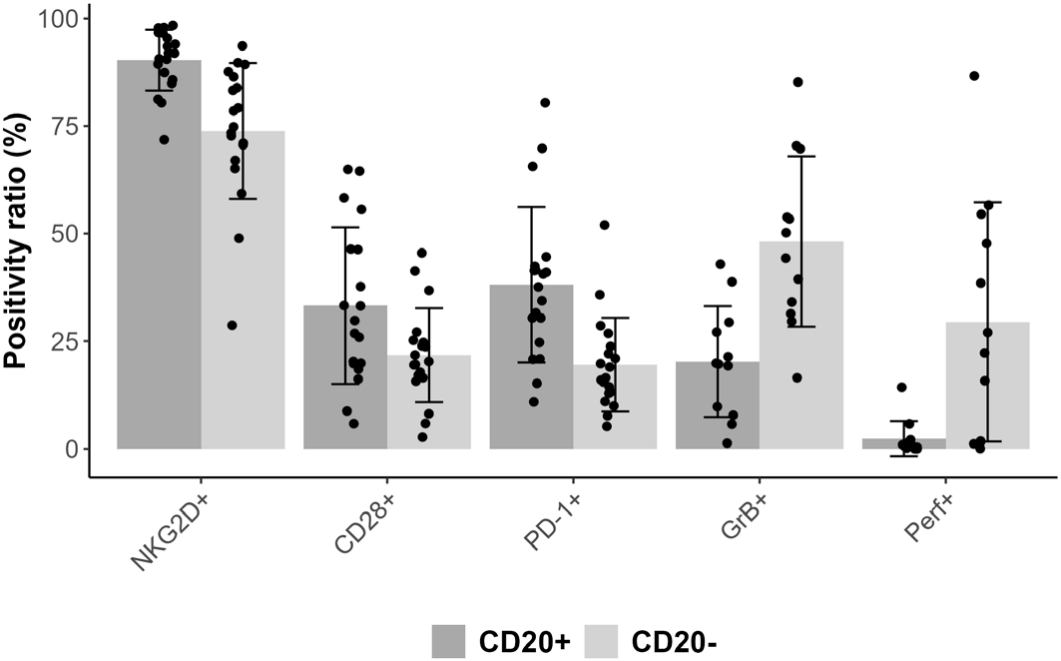
Bar plot representing mean positivity ratio values for each markers combined with error bars of standard deviation and with scatter plot.

**Figure 3:**
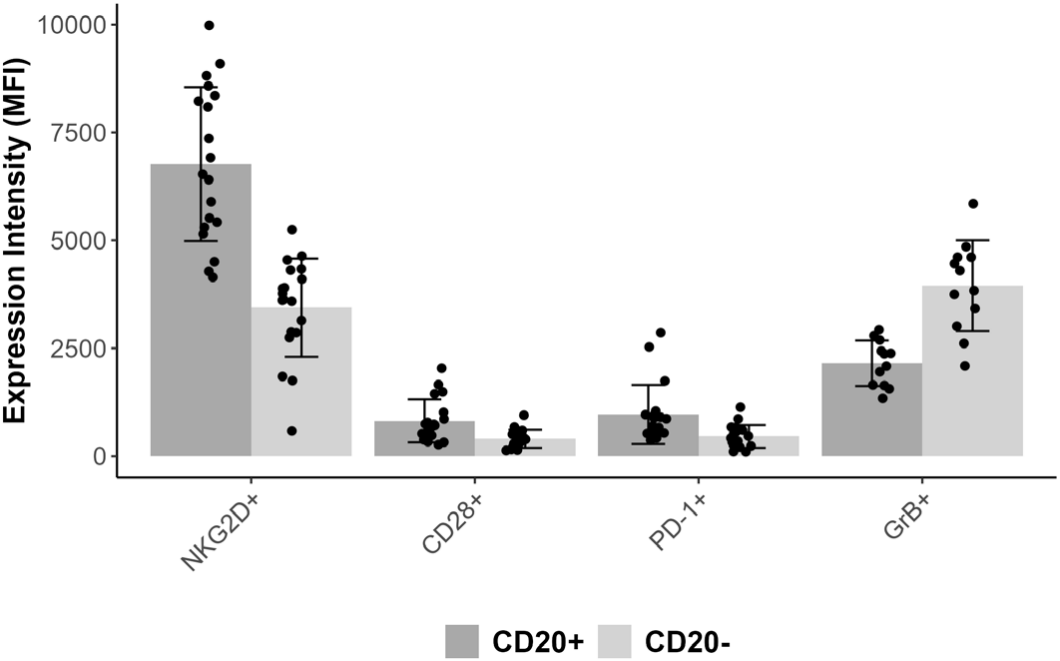
Bar plot representing mean MFI values for each markers combined with error bars of standard deviation and with scatter plot.

**Figure 4:**
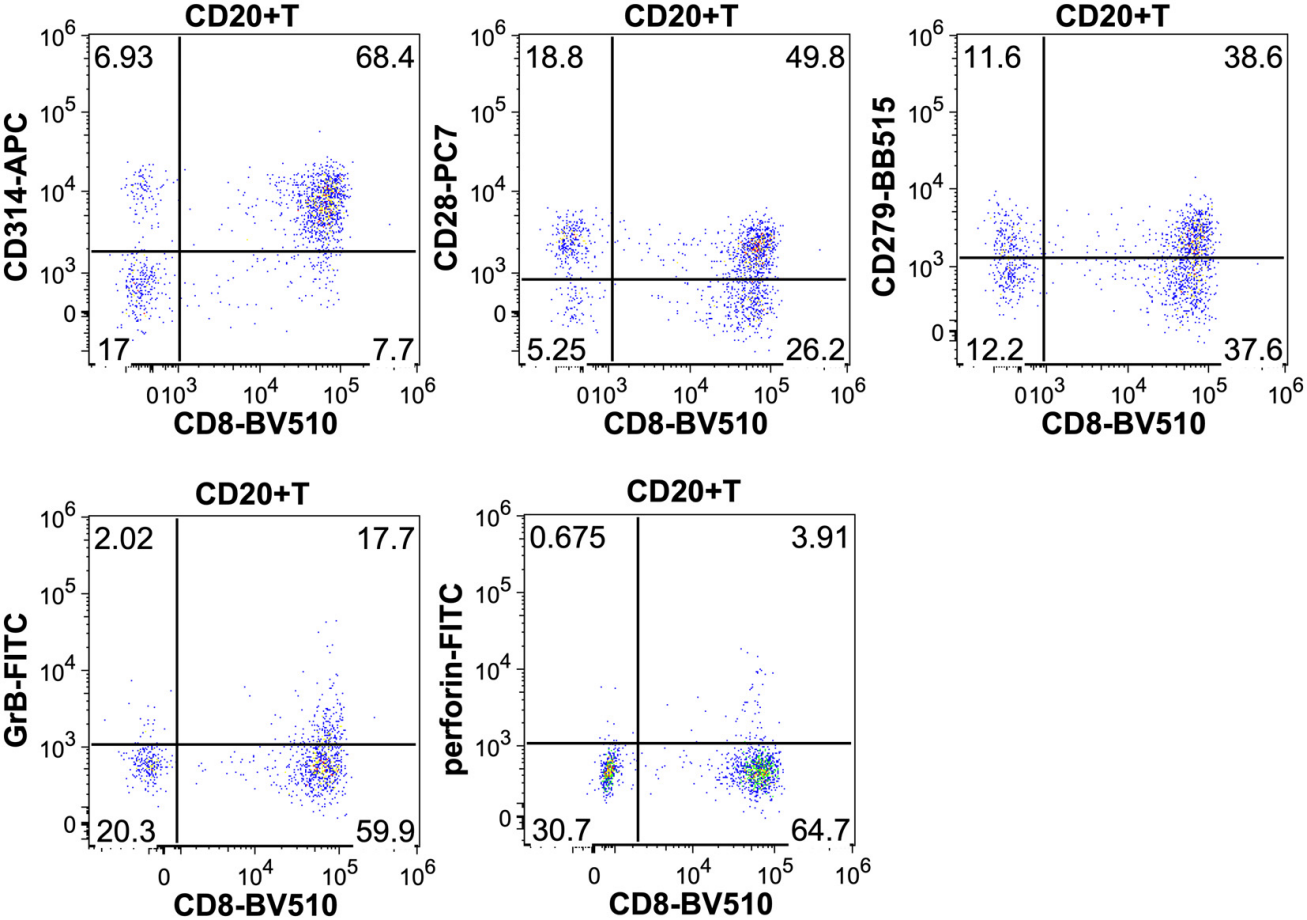
Representative dot plots showing the CD314 (NKG2D), CD28, CD279 (PD-1), granzyme-B and perforin expression of CD8+CD20+ T-cells.

Comparing CD3+CD8+CD20+ and CD3+CD8+CD20- populations we found similarly high (on avereage: 90.3% vs. 73.8%) expression ratios of NKG2D in 19 patients’ samples, however there was a significant difference between the two groups (Wilcoxon signed rank test with continuity correction, p<0.001). The expression level as determined by median fluorescence intensity (MFI) of NKG2D was roughly 2-fold higher on the CD3+CD8+CD20+ subset (on average: 6767 vs. 3441). We examined the expression of CD28 molecule in 19 patients’ samples, where we found significant difference regarding the proportion (on average: 33.3% vs. 21.7%), and in the intensity (on average: 817 vs. 402) of CD28 expression, (p<0.001). Regarding PD-1, our data show differences in respect of expression ratios (on average: 38.1% vs. 19.5%, n=19, p<0.001), as well as MFI values (on average: 965 vs. 457, p<0.001) comparing CD3+CD8+CD20+, and CD3+CD8+CD20- populations, respectively. Regarding granzyme-B and perforin, we found that only a small fraction of CD3+CD8+CD20+ T-cells contained granzyme-B compared to its CD20- counterpart (20% vs. 48%, respectively, n=12, p<0.001). However, CD20+ T-cells express negligible amount of perforin compared to CD20- T-cells (2% vs. 29%, n=12, p<0.002). **Figures 2**, **3** show barplots representing the above mentioned expression data for each marker, while **Figure 4** displays the representative plots of the markers above.

**Figure 5:**
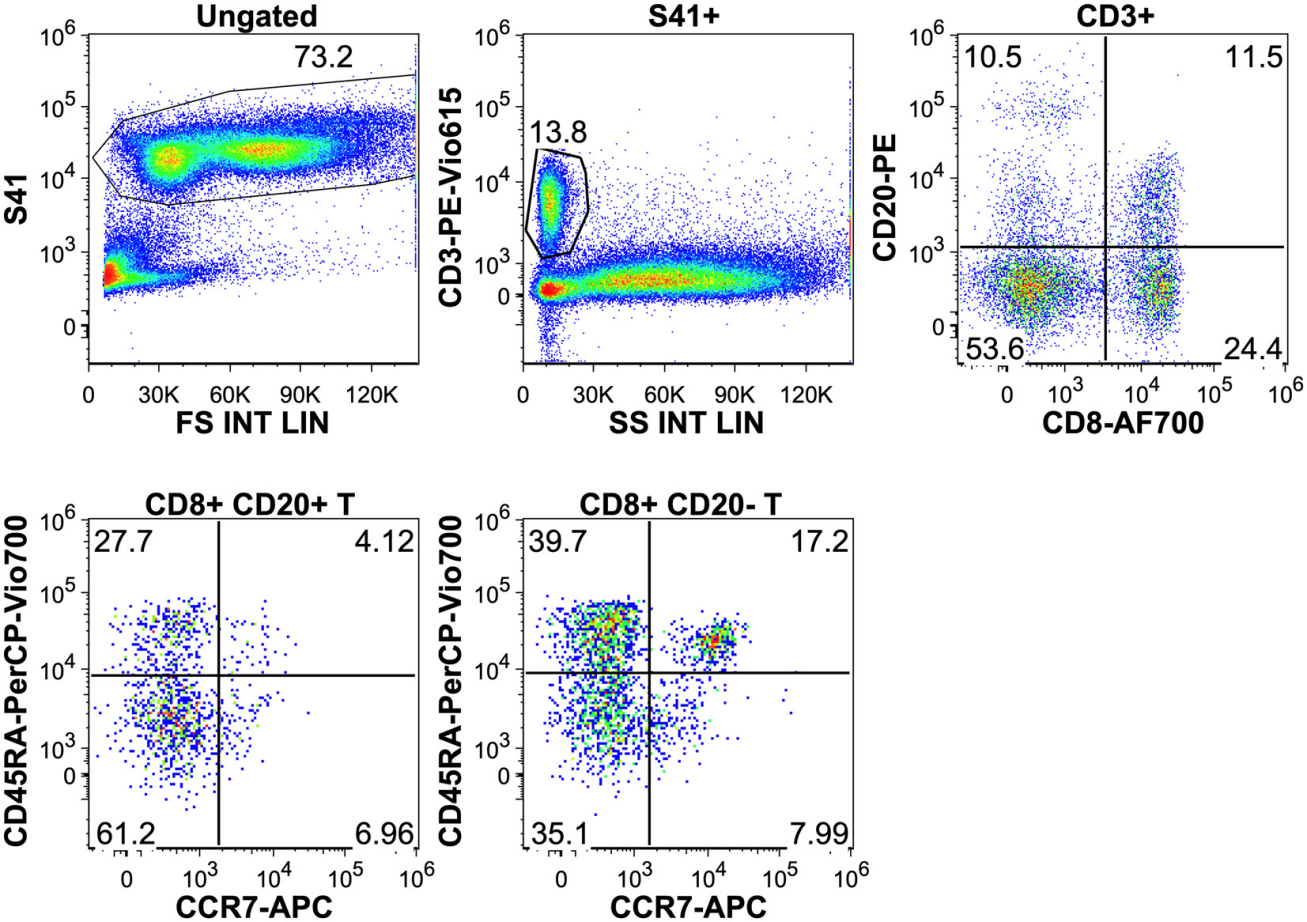
Upper row: gating on CD8+ CD20+ and CD20- T cells. Lower row: distribution of T-cell memory subtypes in the CD20+ and CD20- populations. T-naïve: CCR7+ CD45RA+, T-central memory: CCR7+, CD45RA-, T-effector memory: CCR7-, CD45RA-, T-effector: CCR7-, CD45RA+.

Examining the maturation status of CD8+CD20+ T-cells based on CD45RA and CCR7 expressions, data showed that the 50.2% were effector, 40.1% effector memory, 5.2% central memory and 4.5% naive T-cells. In case of CD8+CD20- T-cells, we found lower ratio of effector memory (41.7%) and higher ratio of naive T-cells (13.5%). **Figure 5** and **Table 1** shows the proportions of the different memory subtypes within CD20+ and CD20- T cells.

**Table 1:** Ratio of T-cell memory subtypes.

	T-naïve %	T-central memory	T-effector memory	T-effector
CD20+ T-cell	4.5%	5.2%	50.2%	40.1%
CD20- T-cell	13.5%	5.8%	41.7%	39.1%

No surface expressions were found regarding CD137, CD25, CD223 and CD103 examining 12 patients’ samples neither on CD3+CD8+CD20+ nor on CD3+CD8+CD20- T-cells.

In the 32 follow-up MM samples, according to statistical data, there was no evidence for a correlation between CD20+ T-cells ratio changes and measurable residual disease ratio by FCM or histology. However, regardless of CD20 expression a positive correlation of surface CD279 expression ratio and proportion of abnormal plasma cell count in bone marrow biopsies was found (Pearson’s product-moment correlation, cor: 0.53, p<0.01 for CD3+CD8+CD20+ and cor: 0.62, p<0.002 for CD3+CD8+CD20- populations).

### 3.2 RNAseq analysis and qPCR of CD20 positive T cells reveal a transcriptionally different population from its CD20 negative counterpart

By performing RNA sequencing on CD3+CD20+ and CD3+CD20- T-cells, we identified numerous differently expressed genes that predominantly associated with T-cell activation, T-cell suppression, and T-cell exhaustion (**Supplementary Figure S1**). Upregulation of CRTAM, CST7, NFKβ2 and downregulation of CTLA4, CD55, NDFIP1, SOCS3, SELL (CD62L) suggest that CD3+CD20+ T-cells may have stronger initial antitumor capability compared to CD3+CD20- T-cells. In contrary we found upregulated (EOMES, TNFSFR9, TIGIT, KLRD1) and downregulated genes (PAG1, GNLY, ANXA1, FGFBP2) that are involved in tumor escape from immune surveillance by suppressing T-cells or by reprogramming T-cells toward T-cell exhaustion. We also found DE genes that have bimodal function on T-cells based on tissue microenvironment e.g.: CDK6, DUSP5, YBX3, MCOLN2, DUSP2. The overexpression of MS4A1 was confirmed via quantitative PCR (qPCR) analysis, demonstrating a 6.5 to 7.6-fold increase in expression in CD20+ cells relative to CD20- cells. This finding aligns with RNA-Seq data, which showed a comparable 6 to 10-fold increase in MS4A1 expression. To ensure robust normalization and accurate comparison, C-Abl was employed as a reference gene, enabling precise quantification of MS4A1 expression differences between the CD20+ and CD20- cell populations (**Supplementary Figure S2**).

## 4 Conclusion

A recently published study precisely summarizes the complex mechanisms of immunoediting in MM through which myeloma cells escape from the control of the immune surveillance, thus becoming one of the worst hematological tumors to treat (20). Immunoediting concept postulates the relationship between the tumor cells and the antitumor responders of the immune system (21). As described, tumor progression consists of 3 phases: elimination, equilibrium and escape. In MM, the multistep evolution from asymptomatic plasma cell dyscrasias (e.g. smoldering myeloma and MGUS) to clinically manifested MM was paralleled with immunoediting phases by Botta et al. (22). Along this line, it was also described that different immune cell actors participate in each phase. Analyzing the flow cytometry results, finding CD28 expression would support that our target cells are involved in the initiation of T-cell activation (23). CD137, being an activation induced costimulatory molecule could indicate that these cells have been recently activated (24). CD25 (25) and CD103 (26) expression could support that these cells are rather involved in regulatory processes. Being positive for NKG2D as a well characterized receptor of cellular distress response, would suggest, that these cells implement antitumor activity (11, 27–29). Producing perforin and granzyme-B would suggest direct cytolytic effect. The panel also contained LAG-3 and PD-1 molecules that implement immune suppression and play a role in T-cell exhaustion (30–33).

Our main hypothesis regarding CD20+ T-cells was that they may play a more competent role in the tumor elimination compared to CD20- T-cells which is supported by the elevated incidence of CD20+ T-cells in MGUS and MM and the expression of CD8, NKG2D, and CD28. Although we found that the antitumor effect does probably not take place through direct cytolysis, since we could detect only a small amount of granzyme-B and a negligible amount of perforin. The higher rate and intensity of surface PD-1 expression on CD20+ T cells in untreated samples and the correlation of the CD8+CD279+ cell ratios with the tumor percentage in follow-up samples suggest that the exhaustion of these T cells may cause tumor progression, however it was found regardless of CD20 expression on T-cells.

The surface phenotype pattern regarding CD45RA and CCR7 of CD20+ T cells suggest that these cells mostly belong to the effector or effector memory subtype. This is further supported by the expression patterns of various genes linked to the development of immunological memory, in particular the downregulation of CCR7, CD45RA, SELL (34), LEF1 (35), FAM65B (36) and IL7R (36) genes and upregulation of NFκB (37). Upregulation of CRTAM that is known to be expressed on activated T-cells (38), and downregulation of CTLA4 (39) CD55 (40), NDFIP1 (41), SOCS3 (42) inhibitory genes suggest antitumor function via regulating T-cell activation pathways. On the other hand, there are significantly upregulated inhibitory genes e.g. TIGIT that interacting with CD155 on antigen presenting cells and on tumor cells also in MM (43), is able to downregulate T and NK cell functions. For that reason, TIGIT is known as a key inhibitor of anti-tumor responses. The upregulated KLRD1 is another inhibitory receptor, previously found on NK cells, that dominantly counteracts T-cell receptor signaling of CD8+ T-effector memory subset (44). Upregulated EOMES and TNFSFR9 and downregulated genes (PAG1, GNLY, ANXA1, FGFBP2) that are involved in tumor escape from immune surveillance by suppressing T-cells or by reprogramming T-cells toward T-cell exhaustion (45–47).

In summary, some of the information in the study confirms previous literary data, but we also offer some novelties in the potential function of CD20+ T-cells. To the best of our knowledge, this study is the first to report the detection of MS4A1 (CD20) as an intracellular transcript on T cells sorted from native bone marrow samples. In the presented research, a larger cohort of bone marrow samples of patients of plasma cell neoplasms were characterized by multiparameter flow cytometric immunophenotyping. Furthermore RNA-sequencing was performed separately on CD20+ and CD20- T-cell subsets providing novel insight into the characteristics and function of these lymphocytes that may play a vital role in immune surveillance and immune escape contributing to progression of myeloma. Deciphering the functions of specific T-cell populations may lead to better understanding of the immune processes of tumor microenvironments and may uncover potential therapeutic targets.

## Data Availability

Original datasets are available in a publicly accessible repository. These data can be found in the European Nucleotide Archive (ENA): https://www.ebi.ac.uk/ena/browser/view/PRJEB83672.
