# Supplementary material for: Multiparameter flow cytometric and transcriptional analyis of CD20 positive T-cells in bone marrow in patients of multiple myeloma and monoclonal gammopathy of undetermined significance
Source: Front Immunol. 2025 Feb 26;16:1464940. doi: 10.3389/fimmu.2025.1464940 (PMC11896981; doi:10.3389/fimmu.2025.1464940)

Supplementary Material

# Supplementary Figure 1.

**Supplementary Figure 1.** The figure shows the heat map clustering of the differentially expressed features for the comparison. Pearson’s metrics has been used in hierarchical clustering of the samples and filtered features.


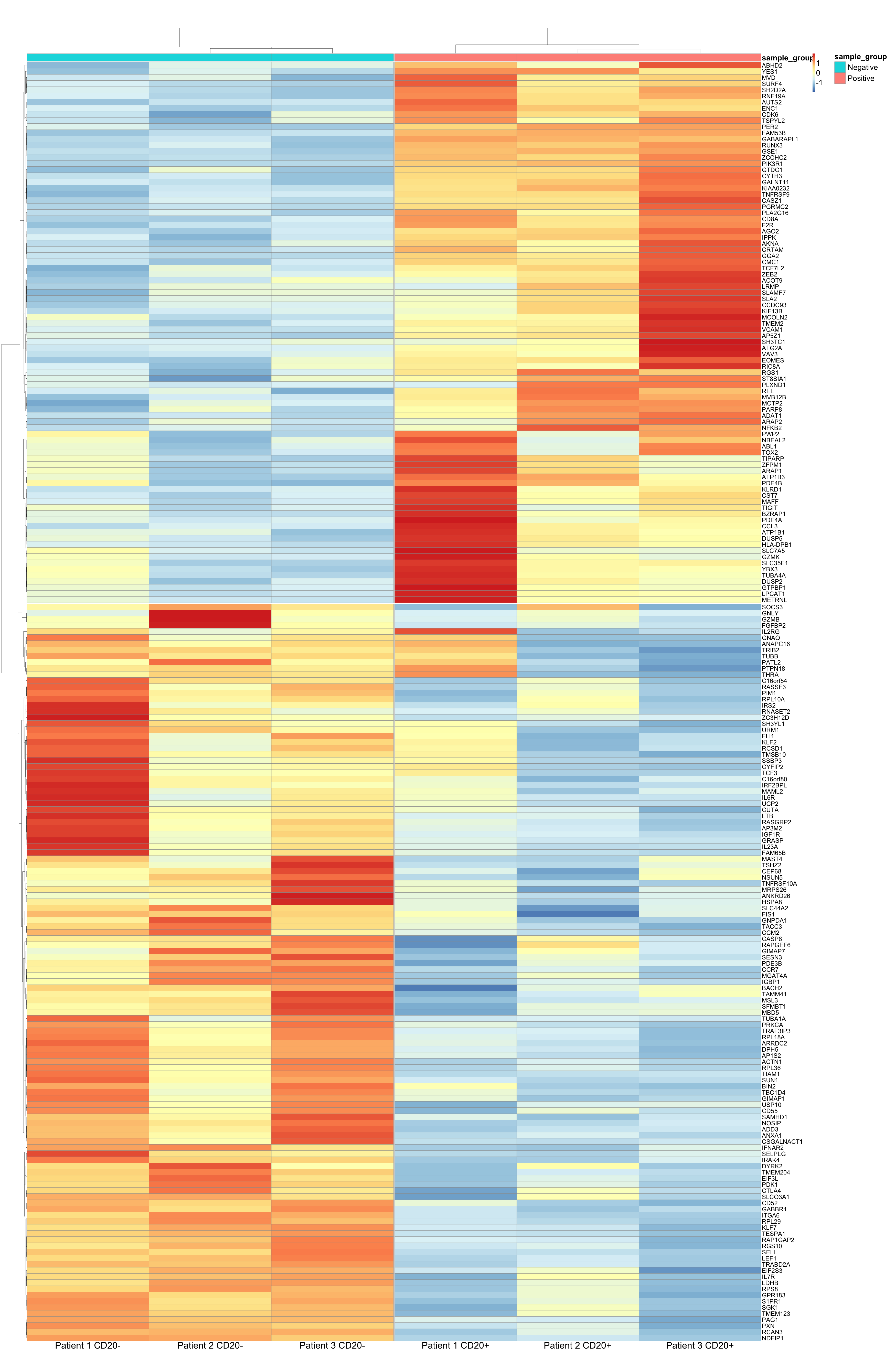


**Supplementary Figure 2.**

**Amplification plot.** Baseline-subtracted fluorescence (y axis) versus number of PCR cycles (x axis). The first two amplification curves represent C-abl (housekeeping gene), the second two amplification curves show MS4A1 (target gene) of CD20+ (red) and CD20- (blue) T-cell populations.


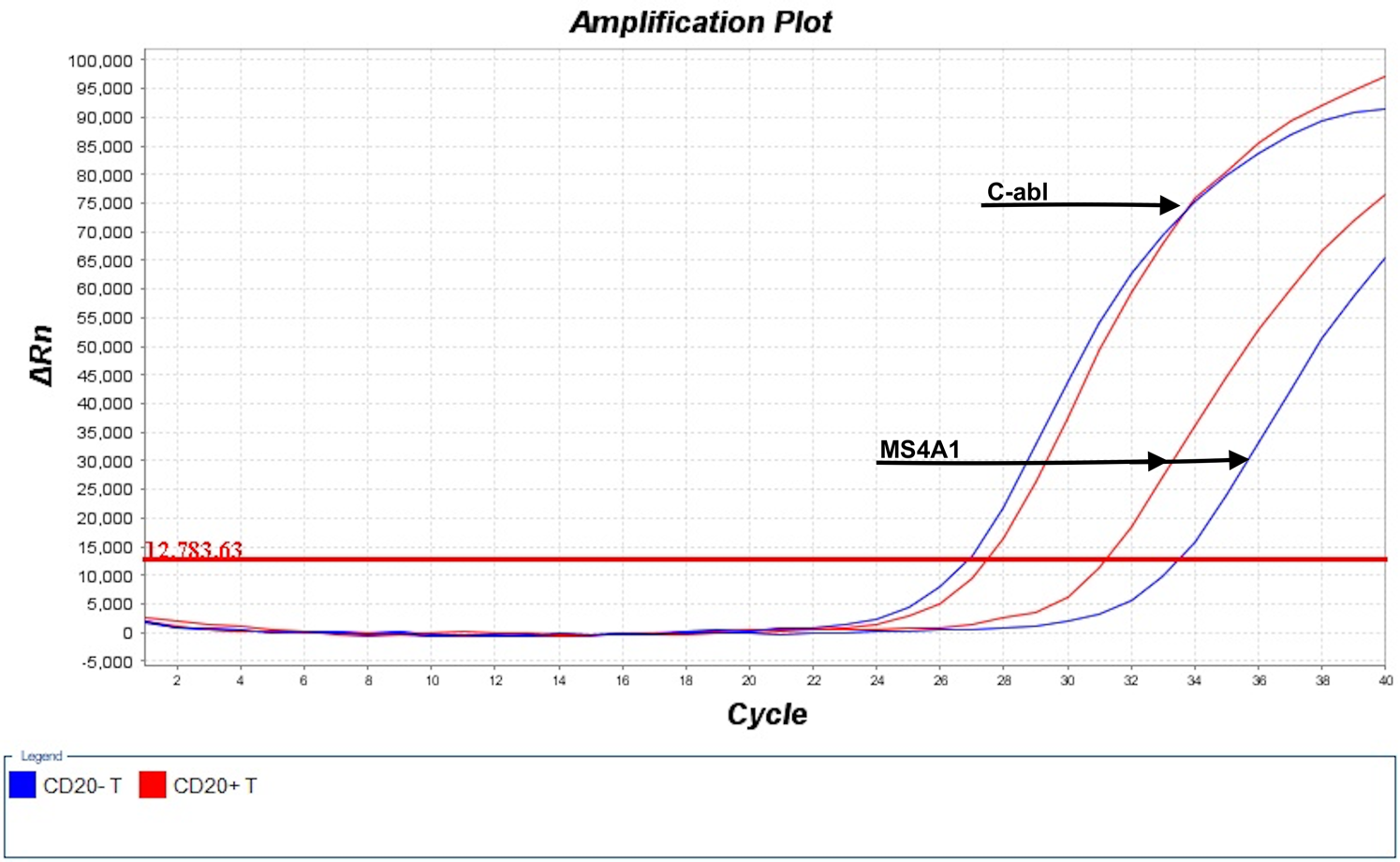

Supplement: Supplementary file 1 [file DataSheet1.zip › Supplementary Figure S1-S2.docx.DOCX]
