# Supplementary material for: Multiparameter flow cytometric and transcriptional analyis of CD20 positive T-cells in bone marrow in patients of multiple myeloma and monoclonal gammopathy of undetermined significance
Source: Front Immunol. 2025 Feb 26;16:1464940. doi: 10.3389/fimmu.2025.1464940 (PMC11896981; doi:10.3389/fimmu.2025.1464940)

Supplementary Material

1 Supplementary Figure 1.

Supplementary Figure 1. The figure shows the heat map clustering of the differentially expressed features for the comparison. Pearson’s metrics has been used in hierarchical clustering of the samples and filtered features.

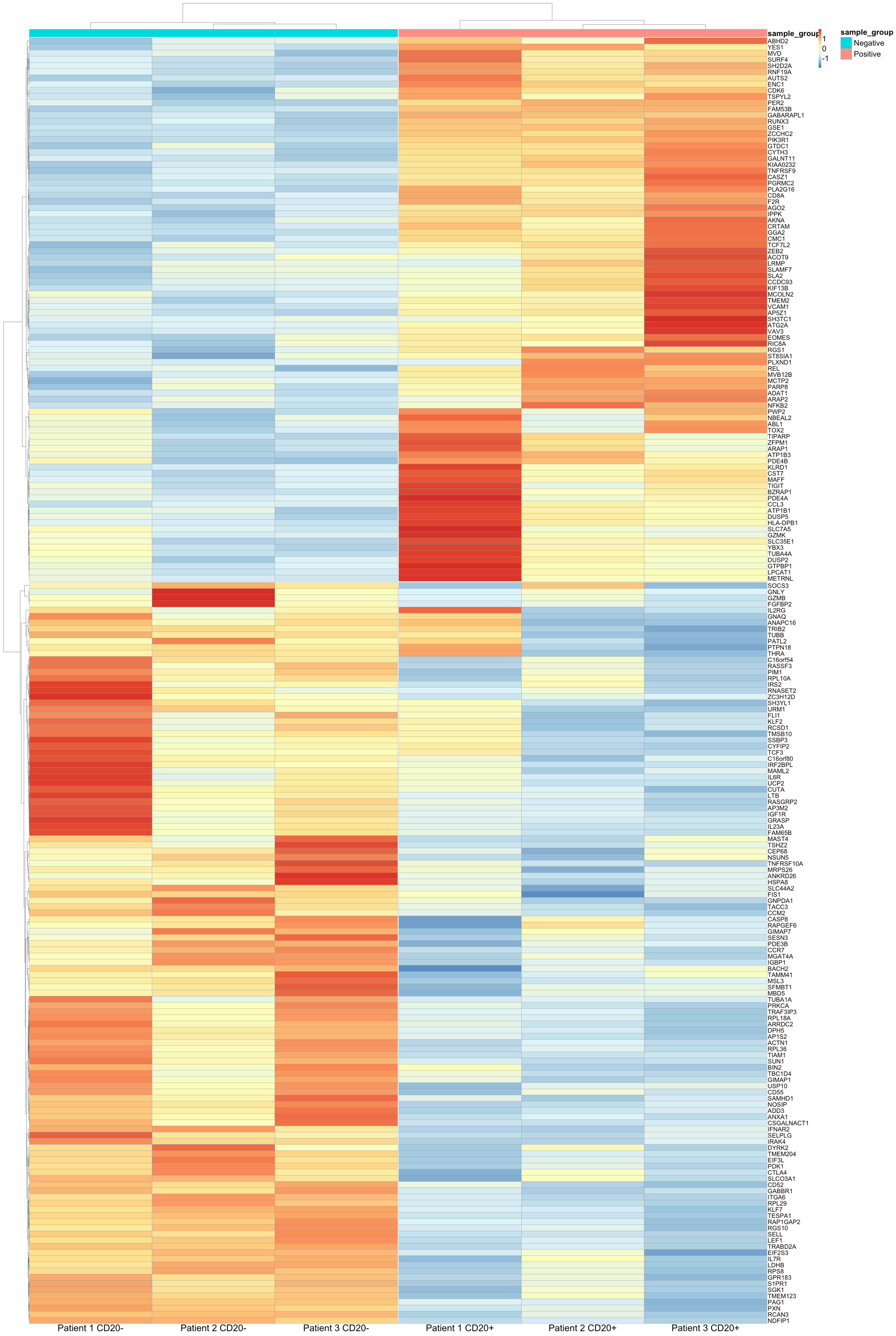
sample_group
1
0
-1
sample_group
Negative
Positive
ABHD2
YES1
MVD
SURF4
SH2D2A
RNF19A
AUTS2
ENC1
CDK6
TSPYL2
PER2
FAM53B
GABARAPL1
RUNX3
GSE1
ZCCHC2
PIK3R1
GTDC1
CYTH3
GALNT11
KIAA0232
TNFRSF9
CASZ1
PGRMC2
PLA2G16
CD8A
F2R
AGO2
IPPK
AKNA
CRTAM
GGA2
CMC1
TCF7L2
ZEB2
ACOT9
LRMP
SLAMF7
SLA2
CCDC93
KIF13B
MCOLN2
TMEM2
VCAM1
AP5Z1
SH3TC1
ATG2A
VAV3
EOMES
RIC8A
RGS1
ST8SIA1
PLXND1
REL
MVB12B
MCTP2
PARP8
ADAT1
ARAP2
NFKB2
PWP2
NBEAL2
ABL1
TOX2
TIPARP
ZFPM1
ARAP1
ATP1B3
PDE4B
KLRD1
CST7
MAFF
TIGIT
BZRAP1
PDE4A
CCL3
ATP1B1
DUSP5
HLA-DPB1
SLC7A5
GZMK
SLC35E1
YBX3
TUBA4A
DUSP2
GTPBP1
LPCAT1
METRNL
SOCS3
GNLY
GZMB
FGFBP2
IL2RG
GNAQ
ANAPC16
TRIB2
TUBB
PATL2
PTPN18
THRA
C16orf54
RASSF3
PIM1
RPL10A
IRS2
RNASET2
ZC3H12D
SH3YL1
URM1
FLI1
KLF2
RCSD1
TMSB10
SSBP3
CYFIP2
TCF3
C16orf80
IRF2BPL
MAML2
IL6R
UCP2
CUTA
LTB
RASGRP2
AP3M2
IGF1R
GRASP
IL23A
FAM65B
MAST4
TSHZ2
CEP68
NSUN5
TNFRSF10A
MRPS26
ANKRD26
HSPA8
SLC44A2
FIS1
GNPDA1
TACC3
CCM2
CASP8
RAPGEF6
GIMAP7
SESN3
PDE3B
CCR7
MGAT4A
IGBP1
BACH2
TAMM41
MSL3
SFMBT1
MBD5
TUBA1A
PRKCA
TRAF3IP3
RPL18A
ARRDC2
DPH5
AP1S2
ACTN1
RPL36
TIAM1
SUN1
BIN2
TBC1D4
GIMAP1
USP10
CD55
SAMHD1
NOSIP
ADD3
ANXA1
CSGALNACT1
IFNAR2
SELPLG
IRAK4
DYRK2
TMEM204
EIF3L
PDK1
CTLA4
SLCO3A1
CD52
GABBR1
ITGA6
RPL29
KLF7
TESPA1
RAP1GAP2
RGS10
SELL
LEF1
TRABD2A
EIF2S3
IL7R
LDHB
RPS8
GPR183
S1PR1
SGK1
TMEM123
PAG1
PXN
RCAN3
NDFIP1
Patient 1 CD20-
Patient 2 CD20-
Patient 3 CD20-
Patient 1 CD20+
Patient 2 CD20+
Patient 3 CD20+

Supplementary Figure 2.

Amplification plot. Baseline-subtracted fluorescence (y axis) versus number of PCR cycles (x axis). The first two curves represent C-abl (housekeeping gene), the second two curves show MS4A1 (target gene) of CD20+ (red) and CD20- (blue) T-cell populations.

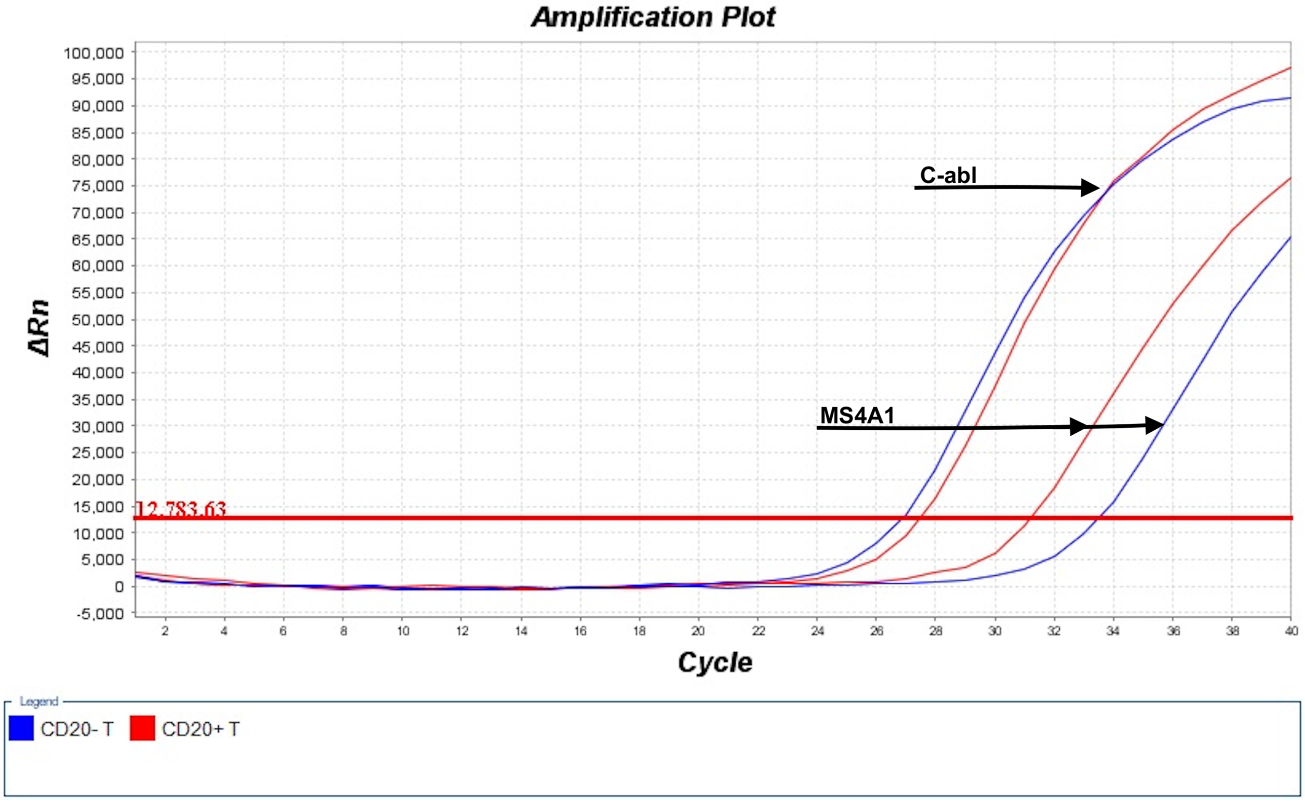

Supplement: Supplementary file 1 [file DataSheet1.zip › Supplementary Figure S1-S2.pdf.PDF]
